# *C. elegans rrf-1* Mutations Maintain RNAi Efficiency in the Soma in Addition to the Germline

**DOI:** 10.1371/journal.pone.0035428

**Published:** 2012-05-04

**Authors:** Caroline Kumsta, Malene Hansen

**Affiliations:** Program of Development and Aging, Sanford-Burnham Medical Research Institute, Del E. Webb Neuroscience, Aging and Stem Cell Research Center, La Jolla, California, United States of America; University Medical Center Groningen, The Netherlands

## Abstract

Gene inactivation through RNA interference (RNAi) has proven to be a valuable tool for studying gene function in *C. elegans.* When combined with tissue-specific gene inactivation methods, RNAi has the potential to shed light on the function of a gene in distinct tissues. In this study we characterized *C. elegans rrf-1* mutants to determine their ability to process RNAi in various tissues. These mutants have been widely used in RNAi studies to assess the function of genes specifically in the *C. elegans* germline. Upon closer analysis, we found that two *rrf-1* mutants carrying different loss-of-function alleles were capable of processing RNAi targeting several somatically expressed genes. Specifically, we observed that the intestine was able to process RNAi triggers efficiently, whereas cells in the hypodermis showed partial susceptibility to RNAi in *rrf-1* mutants. Other somatic tissues in *rrf-1* mutants, such as the muscles and the somatic gonad, appeared resistant to RNAi. In addition to these observations, we found that the *rrf-1(pk1417)* mutation induced the expression of several transgenic arrays, including the FOXO transcription factor DAF-16. Unexpectedly, *rrf-1(pk1417)* mutants showed increased endogenous expression of the DAF-16 target gene *sod-3*; however, the lifespan and thermo-tolerance of *rrf-1(pk1417)* mutants were similar to those of wild-type animals. In sum, these data show that *rrf-1* mutants display several phenotypes not previously appreciated, including broader tissue-specific RNAi-processing capabilities, and our results underscore the need for careful characterization of tissue-specific RNAi tools.

## Introduction

Double-stranded (ds) RNA-induced gene silencing, also called RNA interference (RNAi), was initially discovered in the nematode *Caenorhabditis elegans* as a defense mechanism against viruses and transposable elements. Since then, RNAi has proven to be a powerful experimental tool to gain insight into gene function in multiple organisms [1]. During RNAi in *C. elegans*, dsRNA serves as a trigger for sequence-specific degradation of mRNA as a mechanism of gene silencing [2], [3]. The dsRNA is first cleaved into short-interfering (si) RNAs by a complex containing Dicer, an RNase III family endoribonuclease. These siRNAs are then loaded into the RNA-induced silencing complex (RISC) where they guide recognition of the target-complementary mRNA, which is subsequently cleaved and degraded. Intriguingly, only a few molecules of dsRNA are sufficient to direct degradation of a much larger population of mRNAs [4], [5]. This is due to an amplification process mediated by RNA-dependent RNA polymerases (RdRPs). The RdRPs use siRNAs derived from Dicer-complex processing as primers to produce more dsRNA, with the mature endogenous mRNA as a template. These newly synthesized dsRNA molecules may also feed back into the Dicer complex to be cleaved into additional siRNA (for review, see [4], [5]).

In *C. elegans*, four RdRP homologs have been identified to date: EGO-1 and RRF-1, -2, and -3. EGO-1 and RRF-1 have been implicated in the amplification of exogenous RNAi [6], [7], whereas RRF-3 is required for the production and/or stabilization of a subset of endogenous siRNAs [7], [8]. So far, no clear function has been attributed to the fourth member of the RdRPs, RRF-2. As regulators of the exogenous RNAi pathway, deletion of EGO-1 and RRF-1 leads to RNAi resistance, whereas deletion of RRF-3 results in an increased response to exogenous RNAi, likely due to the reduced competition for resources between the endogenous and exogenous RNAi pathways [8]. Importantly, EGO-1 and RRF-1 have tissue-specific RNAi-processing capabilities. *ego-1* mutants are only partially sensitive to RNAi directed against genes expressed in the germline, but appear fully sensitive to the inactivation of at least one gene, the Notch ligand LAG-2, expressed in the somatic gonad. Moreover, EGO-1 is required for germline development, and is reported to be primarily germline-specific [6]. Based on these findings, the current hypothesis is that EGO-1 is required for the RNAi response targeting germline-specific genes. In contrast, RRF-1 is thought to be required for amplification of the dsRNA signal in the somatic tissues [7]. The known *rrf-1* deletion mutants are viable, fertile, and indistinguishable from wild-type animals. These mutants are sensitive to RNAi against genes expressed in the germline, but are completely resistant to muscle-specific RNAi triggers [7]. Based on these RNAi experiments, the *rrf-1* mutant strain has become the most widely used tool in the *C. elegans* field for determining the site of action of genes specifically in the germline. As an illustration, close to 50 publications to date have used the *rrf-1(pk1417)* allele to conclude that a gene of interest functions in the germline and not in the soma. The initial analysis of the *rrf-1* strain examined muscle-expressed genes as examples of somatic gene expression, and different genes predominantly expressed in the germline [7]. Here, we describe an analysis of somatic tissues besides muscles in which *rrf-1* could function.

Key advantages of using *C. elegans* as a model organism for genetic studies is its experimental tractability while displaying physiological complexity with multiple specialized tissues. The study of tissue-specific functions of genes using RNAi techniques requires robust tissue-selective tools. To address whether the *rrf-1* mutant is an efficient tool for probing the function of genes specifically in the germline, we have characterized the effect of *rrf-1* mutations on processing RNAi in various specialized tissues in *C. elegans.* We confirmed that *rrf-1* mutants are able to process RNAi efficiently in the germline, and are resistant to RNAi in the muscle, as reported previously [7]. However, we found that *rrf-1* mutants are capable of processing RNAi in the intestinal soma against several endogenous genes as well as GFP reporters expressed in the intestine. We also used transgenic reporters to assess the RNAi-processing capabilities of other somatic tissues in *rrf-1* mutants, and found that transgene reduction occurred in a subset of hypodermal cells, the seam cells, whereas the somatic gonad was resistant to *gfp* RNAi. In contrast, the hypodermis was resistant to RNAi against at least two endogenous targets, suggesting that this somatic tissue may be able to process RNAi in some, but not all, cells. Taken together, these results show that the *rrf-1* mutants are sensitive to at least some RNAi triggers in somatic tissues, most notably in the intestine. In addition to imparting an ability to process somatic RNAi, we found that the *rrf-1(pk1417)* mutation also increased the expression of some transgenes in somatic tissues. Lastly, we discovered that the *rrf-1* mutant displayed increased endogenous expression of the FOXO transcription factor DAF-16 target gene *sod-3*, a superoxide dismutase, suggesting that *rrf-1* mutants may have increased DAF-16 activity. While DAF-16 is a key modulator of *C. elegans* longevity, the possible increase in its activity in *rrf-1* mutants does not translate into an extended lifespan or increased stress resistance. Our characterization of the *rrf-1* mutant highlights the need for researchers to exercise caution in using this mutant as a tissue-specific RNAi tool, including in the study of longevity pathways with relevance to the FOXO transcription factor DAF-16.

## Results

### RNAi is Processed in the Intestine of Two Different *rrf-1* Mutants

Because we were interested in using the *C. elegans rrf-1* strain as a tool for tissue-specific RNAi experiments, we sought to characterize the potency of RNAi in various tissues in this mutant. To this end, we employed two different strategies. In the first, we selected several RNAi clones reported to be exclusively or primarily expressed in specific tissues and monitored their corresponding tissue-specific phenotypes in *C. elegans*. We tested these RNAi clones for effects in two different *rrf-1* loss-of-function alleles; *rrf-1(pk1417)* and *rrf-1(ok589)* [7]. The *pk1417* allele contains a large deletion that removes 400 amino acids in the RRF-1 protein, including most of the conserved RdRP-specific residues [7]. The *ok589* allele similarly contains a 303-amino acid deletion in RRF-1, which is also presumed to be a null mutation. As a complementary approach, we crossed several transgenic strains with different tissue-specific GFP expression with the *rrf-1(pk1417)* loss-of-function mutant, and initiated RNAi by feeding these new strains bacteria expressing *gfp* dsRNA. Using these two approaches, we initially reexamined the two tissues that previously had been investigated in *rrf-1* mutants, namely the germline and body-wall muscle [7]. As expected, we found that both *rrf-1* mutants were capable of processing endogenous and *gfp* RNAi triggers in the germline (**Figure S1**) but not in the muscles (**Figure S2**), as shown in [7].

**Figure 1:**
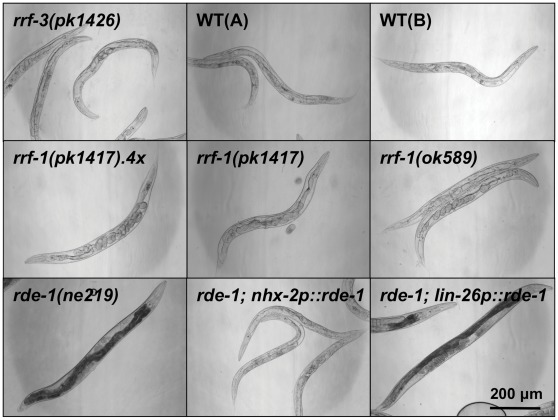
Intestinal *elt-2* RNAi induces prominent phenotypes in *rrf-1* mutants. The indicated *C. elegans* strains were raised on bacteria expressing *elt-2* dsRNA and imaged by DIC microscopy on day 3 of adulthood. The *rde-1* strain and the *rde-1* strain expressing RDE-1 in the hypodermis (*lin-26p::rde-1*) were unaffected by RNAi, whereas all other strains tested show growth defects, a clear appearance, and an abnormal intestine. This experiment was repeated four times with ∼100 worms per strain with similar results. WT: wild-type N2 (A – Hansen lab, B – Tuck lab), *rrf-1(pk1417).4x: rrf-1(pk1417)* outcrossed 4 times to WT(A).

We next tested the ability of the *rrf-1* strain to process RNAi in the intestine. The intestine is the tissue that takes up dsRNA when delivered by ingestion of bacteria, and might therefore be especially sensitive to RNAi by feeding. To test this, we searched for genes that were expressed only in the intestine, and would induce an easily detectable intestinal phenotype upon RNAi-mediated silencing. The GATA-type transcription factor ELT-2 fit these criteria as it is expressed exclusively in the intestine, where it is required for intestinal differentiation [9]. Accordingly, the *elt-2* mutation is known to cause lethality at the L1 stage, with animals displaying a blocked intestinal lumen [9], [10]. Consistent with a role for *elt-2* in the intestine, we found that wild-type *C. elegans* and *rrf-3(pk1426)* mutants (which display enhanced somatic RNAi sensitivity due to a loss-of-function mutation in the RdRP RRF-3 [8]), both experienced constricted and occluded intestines and a clear appearance when subjected to *elt-2* RNAi (**Figure 1**). Moreover, we found that *rde-1(ne219)* mutants (which are resistant to RNAi due to a loss-of-function mutation in the Argonaut protein RDE-1, a key component of the RISC complex [11]) expressing RDE-1 under the intestinal *nhx-2* promoter [12], responded to *elt-2* RNAi similarly to the wild-type animals (**Figure 1**). As expected, a strain expressing RDE-1 in the hypodermis (*rde-1(ne219); lin-26p::rde-1*) was unaffected by *elt-2* RNAi [13] (**Figure 1**). Notably, when we subjected either *rrf-1(pk1417)* or *rrf-1(ok589)* mutants to *elt-2* RNAi, we observed comparable phenotypes to those observed in wild-type animals grown on bacteria expressing *elt-2* dsRNA (**Figure 1**), implying that *rrf-1* mutants are capable of processing *elt-2* RNAi.

We also investigated a second intestinal gene, *pept-1*, which encodes an oligopeptide transporter expressed in the apical membrane of the intestine. Inactivation of *pept-1* abolishes the uptake of peptides into the gut lumen, delays development, and reduces body and brood size [14]. When we subjected wild-type or *rrf-3(pk1426*) animals to *pept-1* RNAi, they displayed a clear body appearance indicative of reduced intestinal fat content [15] (**Figure S3A**), as well as a reduced size (**Figure S3B**). Similar phenotypes were observed in the intestinally rescued *rde-1* strain, as well as in the hypodermally rescued *rde-1* strain (**Figure S3**), suggesting that *pept-1* may be expressed not only in the intestine but also in the hypodermis. Importantly, when *rrf-1(pk1417)* and *rrf-1(ok589)* mutants were subjected to *pept-1* RNAi, they also displayed a clear appearance (**Figure S3A**), and a smaller body size (**Figure S3B**), consistent with *rrf-1* mutants being at least partially capable of processing *pept-1* RNAi in somatic tissues such as the intestine. Collectively, our analysis of endogenous genes indicated that the intestine is capable of processing RNAi in *rrf-1* mutants.

### RNAi is Processed in the Intestine of *rrf-1(pk1417)* Mutants Expressing Different GFP Reporters

**Figure 2:**
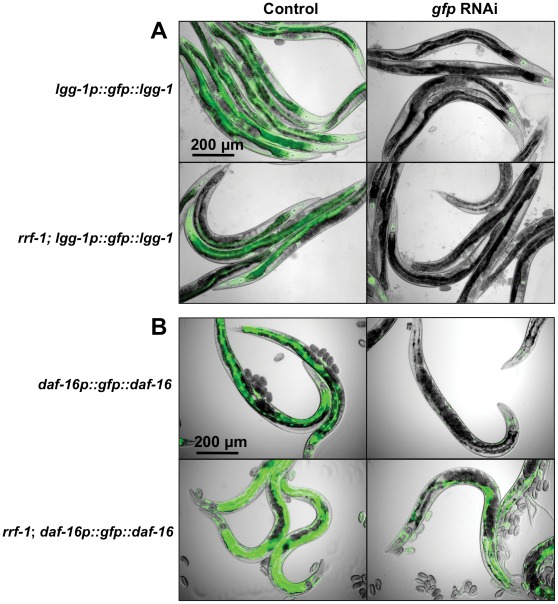
GFP reporters in the intestine of *rrf-1* mutants are repressed by *gfp* RNAi. The indicated *C. elegans* strains were raised on bacteria expressing *gfp* dsRNA, and imaged by DIC and fluorescence microscopy (overlays shown here) on day 1 of adulthood. *rrf-1* mutants carried the *pk1417* allele. (**A**) The *lgg-1p::gfp::lgg-1* reporter is expressed in the intestine, pharynx, somatic gonad, and hypodermis. GFP expression is dramatically reduced upon treatment with *gfp* RNAi in both wild-type and *rrf-1* animals. The exposure time for the GFP channel under control conditions was 15 ms and for *gfp* RNAi 25 ms. In the *gfp* RNAi micrographs, note that the pharynx appears largely unaffected by RNAi in both wild-type and in *rrf-1* animals. See **Figure S5B, D** for additional information on transgene intensity. **(B)** The *daf-16p::gfp::daf-16* reporter is expressed in the intestine, and GFP expression is abolished in the wild-type and reduced in the *rrf-1* background. The *rrf-1* mutant shows visibly higher transgene expression levels compared to the wild-type strain. The exposure time for the GFP channel was 200 ms. See **Figure S7A, B** for additional information on transgene intensity. These experiments were repeated 2–3 times with 30–50 worms per strain and imaging of ∼10 per experiment, with similar results.

To further investigate the finding that *rrf-1* mutants can process RNAi in the intestine, we employed two different transgenic strains with strong GFP expression in the intestine. The first strain overexpresses a GFP-tagged version of the protein LGG-1, a homolog of the mammalian protein LC3, with prominent expression observed in the intestine, pharynx, hypodermis, and somatic gonad [16]. When we fed bacteria expressing *gfp* dsRNA to an *rrf-1(pk1417)* strain carrying the *lgg-1* reporter, we observed a strong reduction in intestinal GFP that was readily apparent by day 1–2 of adulthood (**Figure 2A**). To exclude the possibility that GFP silencing depended on the *lgg-1* transgene, we used a second reporter strain with strong intestinal GFP fluorescence. This strain expresses the FOXO transcription factor DAF-16 with an N-terminal GFP tag, under the control of the *daf-16* promoter [17]. When we subjected the *rrf-1(pk1417)* strain carrying the *daf-16* reporter construct to *gfp* RNAi, this treatment resulted in a noticeable albeit partial loss of intestinal GFP expression compared to wild-type animals (**Figure 2B**). Taken together, our results demonstrate that *C. elegans rrf-1* mutants are at least partially capable of processing RNAi in the intestinal soma.

### The Hypodermis of *rrf-1* Mutants shows Mixed Susceptibility to RNAi

**Figure 3:**
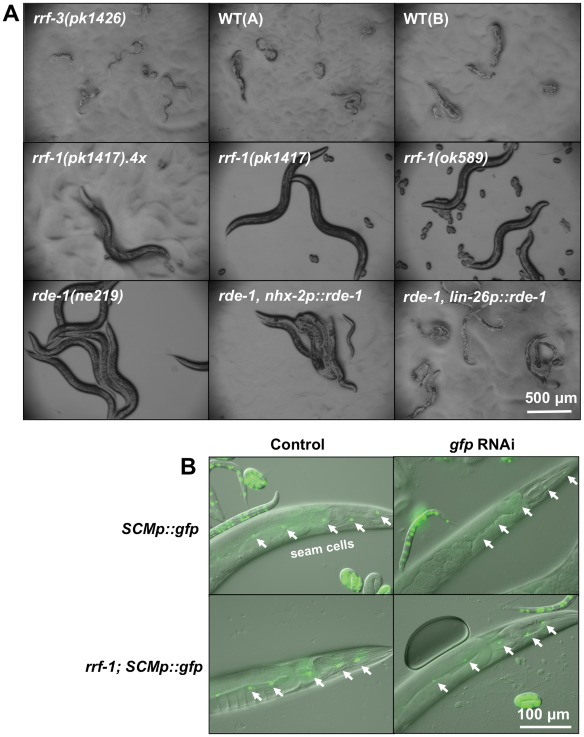
*rrf-1* mutants are resistant to RNAi against the hypodermal gene *bli-3*, but are able to process *gfp* RNAi in seam cells. The indicated *C. elegans* strains were raised on bacteria expressing *bli-3* dsRNA (A) and *gfp* dsRNA (B) and imaged by bright-field or fluorescence microscopy, respectively, on day 1 of adulthood. (A) The *rrf-1* mutants, as well as the RNAi-resistant *rde-1* strain are unaffected by *bli-3* RNAi, whereas all other strains tested show severe molting defects and varying degrees of blister formation. See Figure S4A for additional information. This experiment was repeated three times with 100–200 worms per strain with similar results, also for several generations. WT: wild-type N2 (A – Hansen lab, B – Tuck lab), *rrf-1(pk1417).4x: rrf-1(pk1417)* outcrossed 4 times to WT(A). (B) The *SCMp::gfp* reporter is exclusively expressed in hypodermal seam cells, which are denoted with arrows. GFP expression is abolished in the wild-type background and reduced in the *rrf-1(pk1417)* background. The *rrf-1* mutant shows visibly higher GFP expression levels compared to the wild-type strain. The exposure time for the GFP channel was 100 ms. See Figure S4C for quantification of transgene intensity. These experiments were repeated 3 times with 30–50 worms per strain and imaging of ∼10 per experiment, with similar results.

To determine if other somatic tissues were able to process RNAi in *rrf-1* mutants, we next investigated the hypodermis, a tissue comprised of multiple cell types that establish the basic body form and which secretes the cuticle of the animal. We tested RNAi processing in the hypodermis by inhibiting different genes that are reported to be exclusively or primarily expressed in the hypodermis. The dual oxidase BLI-3 generates hydrogen peroxide required for the crosslinking of collagen dityrosines during cuticle formation [18]. RNAi-induced reduction of the *bli-3* gene, reported to be expressed exclusively in the hypodermis, led to an extreme molting defect in the *rrf-3(pk1426)* strain, in wild-type animals as well as in hypodermally rescued *rde-1* mutants (**Figure 3A**). Interestingly, the intestinally rescued *rde-1* mutant was similarly affected by *bli-3* dsRNA, suggesting that *bli-3 *may be more broadly expressed than reported. Importantly, we observed that the *rrf-1(pk1417)* and *rrf-1(ok589)* strains were largely unaffected by *bli-3* RNAi (**Figure 3A** **and Figure S4A**), even when treated for multiple generations (data not shown). The *rrf-1* strains were similarly resistant to RNAi against another gene reported to be expressed exclusively in the hypodermis, the membrane protein tetraspanin encoded by *tsp-15* [19]. We found that *rrf-1* mutants subjected to *tsp-15* RNAi were resistant to blister formation and defects in body morphology, whereas *tsp-15* RNAi-treated wild-type animals were severely affected (data not shown). In contrast to our analysis of *bli-3* and *tsp-15*, RNAi against two other genes that are largely expressed in the hypodermis, *bli-4* (subtilisin-like serine endoprotease) and *die-1* (zinc finger protein), induced comparable phenotypes in *rrf-1* mutants and wild-type animals (**Figure S4A, B**). However, the interpretation of these results was compromised by the fact that *bli-4* and *die-1* are not exclusively expressed in the hypodermis, but are also present in other somatic tissues including the intestine. Consistent with this notion, we found that both *bli-4* and *die-1* RNAi induced phenotypes in both hypodermally and intestinally rescued *rde-1* strains (**Figure S4B** and data not shown). Taken together, our analysis of endogenous genes does not support an RNAi-processing ability of the hypodermis.

To extend these findings, we analyzed RNAi processing in seam cells, a specialized hypodermal cell type important for the formation of cuticular structures called alae. For these studies, we introduced a seam cell-specific GFP reporter into the *rrf-1(pk1417)* mutant and compared the GFP signal in these mutants and wild-type animals after they had been fed from hatching with bacteria expressing *gfp* dsRNA. Interestingly, we observed that, similar to wild-type animals, *rrf-1(pk1417)* mutants had a significantly reduced GFP signal in their seam cells in response to *gfp* RNAi **(** **Figure 3B** **and Figure S4C)**. Thus, in our transgenic analysis we observed that hypodermal seam cells were at least partially capable of processing *gfp* RNAi, which was in contrast to the results we obtained when analyzing endogenous genes exclusively expressed in the hypodermis. Taken together, these experiments highlight the possibility that a restricted subset of cells in the hypodermis is able to process RNAi.

### The Somatic Gonad is Resistant to RNAi in *rrf-1(pk1417)* Mutants

Because the *rrf-1(pk1417)* mutant has been used in many studies to determine whether a gonadal gene functions in the germline or in the somatic gonad, we were specifically interested in determining if the somatic gonad of *rrf-1* mutants was able to process RNAi. To do this, we examined the persistence of GFP expression after feeding *gfp* RNAi to the *rrf-1(pk1417)* strain carrying a *lag-2p::gfp* transcriptional reporter strain that displays GFP fluorescence in the distal tip cell (DTC) [20]. In the *rrf-1* background, the GFP signal in the DTC persisted upon treatment with *gfp* RNAi, which is consistent with previous reports that somatic tissues of *rrf-1* mutants are resistant to RNAi (**Figure S5A**). Moreover, GFP fluorescence was maintained in the spermatheca and vulva when *rrf-1(pk1417)* animals expressing the *lgg-1p*::*gfp::lgg-1* reporter were treated with *gfp* RNAi (**Figure S5B**). Taken together, these results indicate that the somatic gonad may be resistant to RNAi in *rrf-1* mutants.

### Somatic RNAi Effects in *rrf-1(pk1417)* Mutants are not Due to the Negative RNAi Regulator ERI-6/7

It is possible that the ability of *rrf-1* mutants to process RNAi in at least some somatic tissues is not an intrinsic feature of the *rrf-1* mutation, but instead could be due to mutations in negative regulators of the RNAi pathway in that strain [4]. For example, disruption of the adjacent genes *eri-6* and *eri-7* can lead to an enhanced RNAi response [4]. To exclude the possibility that known *eri-6/eri-7* background mutations have accumulated in the *rrf-1*strains used in this study, we amplified and sequenced the *eri-6/eri-7* locus. Neither of the two *rrf-1* alleles carried the mutation *mg441* (data not shown), a mutation that leads to an enhanced RNAi response due to erroneous splicing and genomic rearrangements in the *eri-6/7* locus (**Figure S6**) [4]. In addition, we designed primers flanking three other reported deletion mutations (*tm1887, tm1716, tm1917*), and found no evidence that the *rrf-1* strains used in this study harbored any of these reported *eri-6/7* mutations (**Figure S6**). While this analysis does not exclude the possibility that unidentified RNAi-enhancing mutations exist in the *rrf-1* mutant, our results are consistent with the *rrf-1* strain possessing an intrinsic ability to process dsRNA in somatic tissues, in addition to the germline.

### *rrf-1(pk1417)* Mutants Display Elevated Expression of Transgenic Arrays

During the course of our experiments with the *daf-16p::gfp::daf-16* reporter strain, we noticed a significantly higher expression of the transgenic array in the *rrf-1(pk1417)* background compared to the wild-type background (**Figure 2B** **and Figure S7A, B)**. This significant increase in GFP fluorescence is likely due to post-translational regulation, since *daf-16* mRNA levels were not significantly elevated in *rrf-1(pk1417)* mutants (**Figure S7C**), consistent with a previous report [24]. Notably, we also observed significantly increased expression of GFP when driven by a seam cell-specific promoter (**Figure 3B** **and Figure S4C**) and by the DTC-specific *lag-2* promoter (**Figure S5C**). These observations suggest that the *rrf-1(pk1417)* mutation can cause a general increase in the expression of transgenic arrays. However, we were not able to detect an increase in transgene expression in *rrf-1(pk1417)* mutants expressing GFP-tagged histone 2B under the germline-specific *pie-1* promoter **(Figure S1B)**, GFP::MYO-3 from the muscle-specific *myo-3* promoter (**Figure S2**), or GFP::LGG-1 from the *lgg-1* promoter **(** **Figure 2A** **and Figure S5D),** suggesting that the *rrf-1* mutation does not have these regulatory effects on all transgenic arrays.

### The Expression of DAF-16/FOXO Target Gene *sod-3* is Elevated in *rrf-1(pk1417)* Mutants

**Figure 4:**
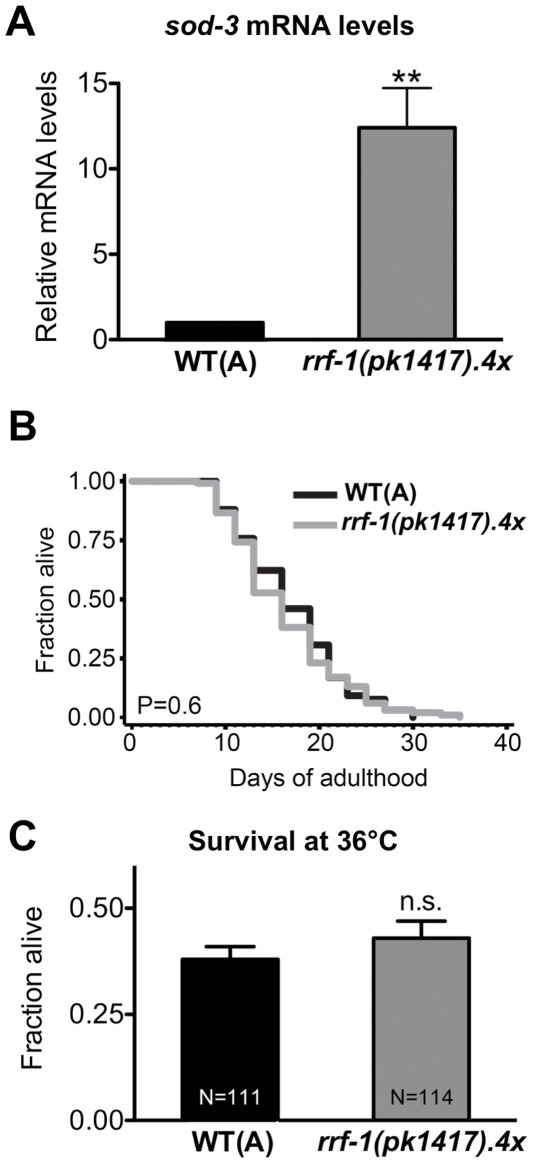
*rrf-1* mutants have increased *sod-3* mRNA levels, a normal lifespan, and normal thermo-tolerance. (**A**) The *sod-3 *mRNA levels of a mixed population of *C. elegans* wild-type (WT, N2(A)) and *rrf-1(pk1417).4x* (*rrf-1(pk1417)* mutant outcrossed four times to WT(A)) strains were determined. Bars show the mean + SEM of three independent experiments; **P<0.005 (Student’s *t*-test)**.** (**B**) Lifespan analysis of wild-type WT(A) and *rrf-1(pk1417).4x* strains at 20°C; P = 0.59, log-rank (Mantel-Cox test). This experiment has been performed three times with similar results; see **Figure S7F** for additional data. (**C**) Thermo-tolerance was measured by assessing survival of WT(A) and *rrf-1(pk1417).4x* strains after 8 h incubation at 36°C; P = 0.36 (Student’s *t-*test). This experiment has been performed three times with similar results.

The FOXO transcription factor DAF-16 plays a major role in modulating longevity in *C. elegans*, most notably through the insulin/IGF-1 signaling pathway [21]. This is likely achieved by the DAF-16–dependent expression of several genes involved in critical biological processes, including stress resistance, metabolism, and disease pathogenesis [21], [22], [23]. Since DAF-16 plays a prominent role in longevity modulation, we investigated the role of *rrf-1* on *daf-16*/FOXO in more detail. Since we found that the *rrf-1(pk1417)* mutation had no effect on *daf-16* mRNA levels (**Figure S7C**), we asked if *rrf-1* mutations affect DAF-16 activity. Superoxide dismutase *sod-3* is a DAF-16 target gene that is often used as a measure of transcriptional activation of DAF-16 [22], [25]. We compared the expression of endogenous *sod-3* levels in wild-type animals and in *rrf-1(pk1417)* mutants by qPCR and unexpectedly, we observed significantly elevated *sod-3* mRNA levels in *rrf-1(pk1417)* mutants compared to wild-type animals (**Figure 4A**). Similarly, we found that *rrf-1(pk1417)* mutants displayed increased expression of a *sod-3p::gfp* transcriptional reporter (**Figure S7D, E)**. While we recognize that the *rrf-1(pk1417)* mutation could directly affect expression of the transgene, as noted above, this result is consistent with the qPCR data measuring endogenous *sod-3* levels. Because DAF-16 is a major transcriptional regulator of *sod-3* expression in *C. elegans*, these observations raise the possibility that DAF-16 activity is elevated in *rrf-1* mutants.

### *rrf-1(pk1417)* Mutants do not show Extended Lifespan or Increased Thermo-tolerance

Increased DAF-16 activity has been associated with longevity in animals with reduced levels of the insulin/IGF-1 like receptor DAF-2 [21], [26]. *daf-2* mutants are long-lived and resistant to various stressors, such as heat and oxidative stress [21], [26]. Since DAF-16 activity may be increased in *rrf-1* mutants, we examined whether *rrf-1* mutations extend lifespan and promote stress resistance in *rrf-1* animals. However, we found no difference between *rrf-1(pk1417)* mutants and wild-type animals in their mean lifespan (**Figure 4B** **and Figure S7**) or their ability to withstand elevated temperature (**Figure 4C**). Thus, although *rrf-1* mutants display increased *sod-3* transcript levels, indicative of elevated DAF-16 activity, these alterations were not sufficient to increase longevity or stress resistance in *rrf-1* mutants.

## Discussion

Here, we have shown that *C. elegans rrf-1* mutants are capable of processing RNAi targeting several genes expressed in the soma (summarized in **Table 1**).

**Table 1:** RNAi efficiency in different tissues in *rrf-1* mutants.

Tissue investigated	Gene or transgene targeted by RNAi	Reported tissue of expression	RNAi phenotype			
			wild-type	*rrf-1*	Observed	Citatio n^1^
Germline	*gld-1*	Germline	**+**	**+**	Pro, Tum	[52]
	*pie-1p::gfp::H2B*	Germline	**+**	**+**	GFP loss	[53]
Muscle	*unc-22*	Muscle	**+**	**−**	Unc/Twitcher	[39]
	*pat-4*	Body-wall muscle, spermatheca, DTC, vulval and anal sphincter muscle, mechanosensory neurons	**+**	**−**	Prz	[54]
	*unc-112*	Body-wall, vulval, spermathecal, uterine, and anal sphincter/depressor muscles	**+**	**−**	Prz	[55]
	*myo-3p::gfp::myo-3*	Body-wall muscle	**+**	**−**	GFP loss	[56]
Intestine	*elt-2*	Intestine	**+**	**+**	Gob, Clr, Lva	[10]
	*pept-1*	Intestine	**+**	**+**	Clr, Gro	[14]
	*bli-4*	Hypodermis, intestine, vulva, ventral nerve cords	**+**	**+**	Bli, Dpy	[28], [57]
	*daf-16p::gfp::daf-16* 2	Intestine, hypodermis, gonad, body-wall muscles, neurons	**+**	**+**	GFP loss	[58]
	*lgg-1p::gfp::lgg-1* 3	Intestine, hypodermis, pharynx, gonad, neurons, body-wall muscles	**+**	**+**	GFP loss	[16]
Hypodermis	*bli-3*	Hypodermis	**+**	**-**	Bli, Dpy	[18]
	*tsp-15* 4	Hypodermis	**+**	**-**	Bli, Dpy	[19]
	*bli-4*	Hypodermis, intestine, vulva, ventral nerve cords	**+**	**+**	Bli, Dpy	[28], [57]
	*die-1*	Embryonically in hypodermis, pharyngeal cells and muscle/gut primordium precursor cells	**+^i^**	**+** 5	Bmd	[59]
	*SCMp::gfp*	Seam cells	**+**	**+/−** 6	GFP loss	[60]
Somatic gonad	*lag-2p::gfp*	Distal tip cell	**+**	**−**	GFP loss	[61]
	*lgg-1p::gfp::lgg-1*	Intestine, hypodermis, pharynx, gonad, neurons, body-wall muscles	**+**	**−**	GFP loss	[16]

‘+’ and ‘−’ refer to the presence or absence of a phenotype in response to RNAi treatment in wild-type or *rrf-1* animals. The *rrf-1(pk1417), rrf-1(pk1417).4x (rrf-1(pk1417) four times outcrossed to WT(A)* and *rrf-1(ok589)* mutants behaved similarly to RNAi targeting endogenous genes and are combined here as *rrf-1*. For strains expressing GFP-tagged transgenes, loss of GFP expression was assayed in response to *gfp* RNAi in the *rrf-1(pk1417)* mutant only.

Phenotype abbreviations: Pro: proximal germ cell proliferation abnormal; Tum: tumorous germline; Prz: paralyzed, Unc/Twitcher: uncoordinated, sub-class, Twitcher; Gob: gut-obstructed; Clr: clear; Lva: larval arrest; Gro: slow growth; Ste: sterile; Bli: blistered; Dpy: dumpy; Bmd: body morphology defects; DTC: distal tip cell.

1 Citations refer to studies describing expression patterns and gene functions of the genes targeted by RNAi and to descriptions of the transgenes used;

2 Only the intestinal expression of *daf-16p::gfp::daf-16* was examined;

3 The intestinal, pharyngeal, and gonadal expression of *lgg-1p::gfp::lgg-1* was examined;

4 Data not shown;

5 RNAi phenotypes observed in 2^nd^ generation;

6 Transgene expression was only partially reduced.

The strongest phenotypic effects were achieved by reducing genes expressed in the intestine. We observed that two different GFP transgenes expressed in the intestine were reduced in *rrf-1(pk1417)* mutants after being exposed to *gfp* RNAi. In addition, we found that RNAi of two endogenous targets, *elt-2* and *pept-1*, induced robust phenotypes in two different *rrf-1* mutants. While *elt-2* RNAi seemed to play an exclusively intestinal-specific role, *pept-1* RNAi induced effects in both intestinally and hypodermally rescued *rde-1* strains [12], [13]. This result may indicate that *pept-1* functions as an oligopeptide transporter not only in the intestine, but also in the hypodermis. Alternatively, we note the possibility that the rescued *rde-1* strains are not as specific in their RNAi sensitivity as anticipated.

We also observed RNAi-processing capabilities in another somatic tissue, the hypodermis, albeit to a limited extend. We found that *rrf-1* mutants expressing a transgenic reporter in a specific subset of hypodermal cells, i.e., the seam cells were susceptible to knockdown of *gfp*. In contrast, *rrf-1* mutants failed to respond to RNAi against *bli-3* or *tsp-15*, which are both genes reported to be expressed exclusively in the hypodermis. Moreover, we found that RNAi against additional genes with hypodermal expression (*bli-4* and *die-1*) induced similar phenotypes in both wild-type animals and *rrf-1* mutants. While the phenotypes induced by *bli-4* and *die-1* RNAi in *rrf-1* mutants could originate from the knockdown of these genes in other somatic tissues, including the intestine (**Table 1**), it seems that the hypodermis of *rrf-1* mutants is not generally capable of processing dsRNA triggers. Instead, our findings suggest that either a restricted subset of cell types in the hypodermis of *rrf-1* mutants is capable of processing RNAi, or perhaps only some endogenous targets can be degraded. Further analysis of endogenous targets, especially expressed in hypodermal seam cells is warranted to address this possibility.

Taken together, our analysis indicates that *rrf-1* mutants can process RNAi in the soma, most notably in the intestine, demonstrating that *rrf-1* mutants have broad RNAi-processing capabilities. Consistent with this notion, new information on the expression pattern of the predominantly germline-specific genes *pos-1*, *par-1* and *pop-1* originally tested by Sijen et al. [7], suggests that these genes are also expressed in somatic tissues [27], [28], [29], [30], which further corroborates an RNAi-processing role in the soma of *rrf-1* mutants.

These findings have important implications for the use of this strain in tissue-specific RNAi studies, as well as for the interpretation of published reports using RNAi in this strain. Our literature search revealed that close to 50 studies used the *rrf-1(pk1417)* mutant to investigate the role of a gene of interest in the germline versus somatic tissues. However, several of these publications used additional and alternative methods to confirm a germline requirement for the gene of interest, such as mosaic analysis [31], [32], [33], [34], [35]. Indeed, most of the published studies used *rrf-1(pk1417)* mutants to determine if a gene expressed in the gonad has a specific role in the germline or the somatic gonad [36], [37], [38]. Our studies indicated that the *rrf-1* mutant is resistant to RNAi in the somatic gonad by using two different *gfp*-tagged reporters. Specifically, we found that the GFP signal was maintained in the vulva and spermatheca of an *rrf-1; lgg-1p::gfp::lgg-1* reporter strain, as well as in distal tip cells of an *rrf-1; lag-2p::gfp* reporter strain (**Table 1**). Ideally, these experiments should be complemented by an analysis of endogenous genes; however, we have been unsuccessful in inducing phenotypes in wild-type animals using RNAi against the endogenous genes we investigated, including *lag-2* (data not shown). Based on our results, the *rrf-1* strain may thus be a useful tool for determining a differential requirement for a gene in the germline and somatic gonad.

RRF-1 is one of two RdRPs involved in the exogenous siRNA pathway that amplifies dsRNA triggers, and has been reported to act in the soma of *C. elegans* [7]. In contrast, the second RdRP, EGO-1, is thought to be required for processing of siRNA targeting germline-specific genes [6]. Our observation that the *rrf-1* mutant is capable of processing RNAi in somatic tissues could suggest the existence of unidentified enzymes that have overlapping functions with RRF-1, or suggest a role for EGO-1 outside of the germline. Although Smardon et al. have reported that EGO-1 expression is primarily germline-specific [6], it may also be expressed in somatic tissues. Moreover, germline EGO-1 activity may be sufficient to produce enough siRNA to affect other tissues. Alternatively, RRF-2 or an unidentified RdRP may play roles in processing RNAi in the absence of RRF-1.

One of the striking features of RNAi in *C. elegans* is its systemic nature, in which the dsRNA spreads from the tissue of uptake, most often the intestine, into other tissues [39], [40], [41], [42]. In our experiments, the dsRNA trigger was delivered by feeding the animals bacteria expressing dsRNA. Therefore, it is possible that sufficient dsRNA was ingested to at least partially knock down the targeted mRNA in the intestine without the need for an RdRP-mediated amplification reaction, making the mechanism RdRP-independent. This may also be the case for the worm pharynx, which processes bacteria upon intake. Although we have attempted to directly address a role for the pharynx using endogenous genes as RNAi targets, these experiments have been hampered by the apparent refractory nature of the pharynx to RNAi (see **Figure 2A**, and data not shown). While our objective in this study was to characterize the *rrf-1* strain for processing of RNAi delivered by feeding, future studies should determine if direct injection of the dsRNA into the germline have the same effect on somatic gene expression of *rrf-1* mutants, including in the intestine. Perhaps direct delivery of dsRNA to the germline would reduce exposure of the intestine and other tissues to dsRNA and might even prevent the degradation of mRNAs encoded by intestinal-specific genes. These additional tissues may include ones we have not yet assayed, for example neurons, which are generally considered refractory to RNAi by feeding [43], [44].

Unexpectedly, we observed that *rrf-1* mutants displayed increased expression of an important target gene of the FOXO transcription factor DAF-16, the superoxide dismutase SOD-3. Since DAF-16 is a major regulator of *sod-3,* this observation raises the possibility that *rrf-1* mutants have increased activity of DAF-16. DAF-16 activity is required for the extended longevity of many *C. elegans* mutants, including *daf-2* mutants [21]. In *daf-2* mutants, these phenotypes are mediated by nuclear translocation and activation of DAF-16, which in turn causes upregulation of target genes such as *sod-3*. We did not, however, observe nuclear localization of DAF-16 in the *rrf-1* background (**Figure 2A** **and Figure S7A**, and data not shown). Additionally, we found no increase in longevity or thermo-tolerance in the *rrf-1(pk1417)* mutant, suggesting that if *rrf-1* mutants have increased DAF-16 activity, it is not sufficient to extend lifespan. This would be consistent with the observation that increased expression of SOD-3 does not extend *C. elegans* lifespan [45]. It remains to be explored how inhibition of the RdRP *rrf-1* leads to increased expression of SOD-3, and whether other DAF-16 targets are similarly affected. Regardless of the exact mechanism, our novel findings that *rrf-1* mutants show efficient RNAi in somatic tissues and also exhibit increased expression of the DAF-16-regulated stress response gene *sod-3,* should be considered before using these mutants in tissue-specific RNAi studies, especially in the study of longevity pathways with relevance to the FOXO transcription factor DAF-16.

In addition to observing a new somatic RNAi-processing ability and increased expression of endogenous *sod-3*, we also noted that multiple, but not all, transgenes used in our investigation were expressed at higher levels in *rrf-1* mutants. These observations suggest that RRF-1 can negatively regulate transgene expression, at least in some cases. Notably, we observed no direct correlation between the tissues in which transgenic expression was increased by *rrf-1* mutation and the cell types that responded to RNAi processing. Moreover, the *daf-16p::gfp::daf-16* and *lgg-1p::gfp::lgg-1* reporters both responded to *gfp* RNAi in the intestine when expressed in *rrf-1* mutants, but only the *daf-16p::gfp::daf-16* reporter was notably upregulated by the *rrf-1* mutation. This suggests that the mechanism regulating transgene expression in *rrf-1* mutants is not identical to that of RNAi processing. While the mechanism of transgene regulation is currently unclear, it is an important result because the increased expression of specific transgenes in *rrf-1* mutants could induce morphological or cellular changes. Indeed, *rrf-1* mutants may display many more phenotypes than noted previously or reported here, and this information should be kept in mind when using this mutant as a tool for studying gene function.

## Materials and Methods

### Strains and Culture Conditions

Strains were maintained and cultured under standard conditions at 20°C using *E. coli* OP50 as a food source [46], except when subjected to RNAi treatment. We used these previously reported strains in this study (see **Table 1** for references):

WT(A): Hansen-lab N2, WT(B): Tuck-lab N2 (Simon Tuck, University of Umeå), NL2098: rrf-1(pk1417) I, RB798: rrf-1(ok589) I, NL2099: rrf-3(pk1426) II,

WM27: rde-1(ne219) V,

VP303: rde-1(ne219) V; kbIs7[nhx-2p::rde-1 + rol-6(su1006)],

NR222: rde-1(ne219) V; kbIs9[lin-26p::nls::gfp, lin-26p::rde-1 + rol-6(su1006)],

DA2123: adIs2122[lgg-1p::gfp::lgg-1 + rol-6(su1006)],

CF1934: daf-16(mu86) I; muIs109[daf-16p::gfp::daf-16 + odr-1p::rfp],

AZ212: unc-119(ed3) III; ruIs32[unc-119(+) pie-1(+) pie-1p::gfp::H2B],

RW1596: myo-3(st386) V; stEx30[myo-3p::gfp::myo-3 + rol-6(su1006)],

CF1553: muIs84[sod-3p::gfp],

JK2049: qIs19[lag-2p::gfp + rol-6(su1006)] V,

JR667: unc-119(e2498::Tc1) III; wIs51[SCMp::gfp + unc-119].

We note that the promoter of the seam-cell marker (SCM) used for GFP expression in the JR667 strain has not been reported.

We created the following new strains for this study:

MAH19: rrf-1(pk1417) I, myo-3(st386)V; stEx30[myo-3p::gfp::myo-3 + rol-6(su1006)],

MAH22: rrf-1(pk1417) I; adIs2122[lgg-1p::gfp::lgg-1 + rol-6(su1006)],

MAH23: rrf-1(pk1417) I outcrossed 4 times to Hansen-lab N2 and referred to as rrf-1(pk1417).4x,

MAH74: rrf-1(pk1417) I; unc-119(ed3) III; ruIs32[unc-119(+) pie-1(+) pie-1p::gfp::H2B],

MAH97: rrf-1(pk1417) I; muIs109[daf-16p::gfp::daf-16 + odr-1p::rfp],

MAH99: rrf-1(pk1417) I; muIs84 [sod-3p::gfp],

MAH132: rrf-1(k1417) I; unc-119(e2498::Tc1) III; wIs51[SCMp::gfp + unc-119],

MAH133: rrf-1(pk1417) I; qIs19[lag-2p::gfp + rol-6(su1006)] V.

We note that we observed germline defects in 5–10% of the assayed animals of the *rrf-1(pk1417); lag-2p::gfp* strain (data not shown), whereas no defects were observed when this transgene was expressed in the wild-type background, or in the *rrf-1(pk1417)* mutant without expression of the *lag-2p::gfp* reporter, or in the *rrf-1(pk1417); pie-1p::gfp::H2B* strain (data not shown).

### RNAi Treatment and Imaging

The *gfp* RNAi clone was obtained from Dr. Andrew Dillin (Salk Institute). The *bli-4* and *elt-2* clones used were obtained from the Vidal RNAi library [47]; the *elt-2, pept-1, pat-4, unc-112, pha-4, gld-1* and *bli-3* RNAi clones were obtained from the Ahringer library [48]. All RNAi clones were verified by sequencing. For the RNAi treatment, worms were synchronized by hypochlorous acid treatment and the eggs seeded on the NGM plates with bacteria expressing the dsRNA. Differential interference contrast (DIC) microscopy was performed on a Zeiss Imager Z1 after mounting worms on a 2% agarose pad containing 0.1% NaN_3_, in M9 medium also containing 0.1% NaN_3_. Worms on plates were imaged on plates with a Leica DFC310 FX camera. For the analysis of GFP expression, fluorescence images were collected on a Zeiss Imager Z1 at the exposure times indicated in the figure legends. Image analysis was performed with ImageJ software (National Institutes of Health), by tracing the intestine (for strains expressing *daf-16p::gfp::daf-16* and *sod-3p::gfp*) or the entire worm (*lgg-1p::gfp::lgg-1* expressing strains) and measuring the integrated density. The analysis of strains expressing *lag-2p::gfp* was performed by tracing a defined circular DTC-encompassing region of interest and measuring the mean density. For the analysis of the GFP intensity of the seam cells, 1–5 regions of interest encompassing 2360 pixels were drawn around the single seam cell bodies and the average mean density per worm was calculated. For the analysis of the germline overproliferation following *gld-1* RNAi treatment, worms were washed off the plates on day 3 of adulthood and washed three times in M9 media before fixing in methanol overnight at -20°C. For DAPI staining, methanol was removed by washing with M9 media, 0.1 µg/ml DAPI in M9 was added, and samples were incubated for 20 min on a slow nutator. Worms were washed with M9 and then mounted on agarose pads for imaging. Statistical analyses were performed by Student’s *t-*test and two-way ANOVA using GraphPad Prism (La Jolla, CA). If RNAi was induced for a second generation, the eggs of the first generation of animals were transferred to new RNAi plates and these animals were then scored. All experiments were carried out at 20°C.

### Quantitative RT-PCR

The graphs show the average from three biological replicates (with three technical replicates). For each experiment, total RNA was isolated from a mixed population of ∼2000 nematodes that were flash frozen in liquid nitrogen. RNA was extracted using Trizol (Life Technologies, Grand Island, NY) and purified using the Qiagen RNeasy kit (Valencia, CA), including an additional DNA digest step using the Qiagen Dnase I kit. The M-MuLV reverse transcriptase and random 9-mer primers (New England Biolabs, Ipswich, MA) were used for reverse transcription of 1 µg of RNA per sample [49]. Quantitative PCR was run using SYBR Green Master Mix in a Roche LC480 LightCycler (Indianapolis, IN). A standard curve was run for each primer on serial dilutions of a mixture of different cDNAs, and the observed C_T_ values were converted to relative values according to the primers’ standard curves. The mRNA levels of target genes were normalized to those of the housekeeping genes cyclophilin (*cyn-1)* and nuclear hormone receptor *nhr-23*. Primer information is available upon request.

### Lifespan Analysis

Lifespan was measured at 20°C with at least 90 worms in each experiment [50]. Animals were scored as dead when they failed to respond to gentle prodding with a platinum wire pick. Censoring occurred if animals desiccated on the edge of the plate, escaped, ruptured, or suffered from internal hatching. For statistical analysis Stata software was used (StataCorp, College Station, TX). P values were calculated with the log-rank (Mantel-Cox) method.

### Thermo-tolerance Assay

The survival of *C. elegans* at elevated temperatures was measured after an 8 h incubation at 36°C [51]. The synchronized wild-type and *rrf-1(pk1417)* animals were transferred to assay plates on day 1 of adulthood and the thermo-tolerance experiment was performed on day 3 of adulthood. This experiment was performed at least 3 times with similar results. Student’s *t*-test was used to calculate P values.

### PCR Assay for *eri-6/7* Mutations and Genomic Rearrangement

Single worms were used in these PCR assays. Worms were lysed in 10 µl proteinase K buffer (1 µg/µl proteinase K, 1x PCR buffer, H_2_O) at 65°C for 1 h and 95°C for 15 min. Taq polymerase was used to amplify the PCR product for 35 cycles with an annealing temperature of 58°C. Primer sequences were: P1 (MH439): 5′-GCCAGTGCTTCACGTGTC-3′, P2 (MH440): 5′-CGAAGCCCAGATGTGAGATC-3′ P3 (MH479): 5′-TAAGTTTTCCTAAAATGATTTC-3′. This protocol was adapted from [4].

## Supporting Information

Figure S1 ***rrf-1*** **mutants are capable of processing RNAi in the germline.** (**A**) The indicated *C. elegans* strains were raised on bacteria expressing *gld-1* dsRNA, fixed in methanol on day 3 of adulthood, and subsequently stained with DAPI and imaged by fluorescence microscopy. All strains display mitotic germ cell overproliferation except the RNAi-resistant *rde-1* strain, which shows typical mitotic and meiotic zones in the distal end of the gonad, and eggs in utero in the proximal part of the gonad. This experiment was repeated twice with 50–100 worms per strain with similar results. WT: wild-type N2 (A – Hansen lab, B – Tuck lab), *rrf-1(pk1417).4x: rrf-1(pk1417)* outcrossed 4 times to WT(A). (**B**) The indicated *C. elegans* strains were raised on bacteria expressing *gfp* dsRNA and imaged by DIC and fluorescence microscopy (overlays shown here) on day 1 of adulthood. *rrf-1* mutants carried the *pk1417* allele. The *pie-1p::gfp::*H2B reporter is expressed in the germline of *C. elegans*. GFP expression is completely abolished upon treatment with *gfp* RNAi, in both the wild-type and *rrf-1* backgrounds. The image shows one arm of the gonad including oocytes, and was acquired with an exposure time of 100 ms. This experiment was repeated twice with 30–50 worms per strain and imaging of ∼10 per experiment, with similar results.
(TIF)

Figure S2 ***rrf-1*** **mutants are resistant to RNAi in the muscle.** (**A**) The indicated *C. elegans* strains were raised on bacteria expressing *gfp* dsRNA, and imaged by fluorescence microscopy on day 1 of adulthood. *rrf-1* mutants carried the *pk1417* allele. The body-wall muscle-specific GFP expression is maintained in the *rrf-1* background, but completely abolished in the wild-type background. The exposure time for the GFP channel was 200 ms. This experiment was repeated twice with similar results. (**B**) The indicated *C. elegans* strains were raised from hatching on bacteria expressing muscle-specific *unc-112*, *pat-4,* and *unc-22* RNAi and were assayed for paralysis and twitching on day 1 of adulthood. The experiments have been repeated at least two times with similar results. WT: wild-type N2 (A – Hansen lab, B – Tuck lab), *rrf-1(pk1417).4x: rrf-1(pk1417)* outcrossed 4 times to WT(A). Phenotype abbreviations: Prz: paralyzed; Unc: uncoordinated, sub-class, Twitcher. n.a.: not assayed.
(TIF)

Figure S3 **Intestinal** ***pept-1*** **RNAi induces phenotypes in** ***rrf-1*** **mutants.** The indicated *C. elegans* strains were raised on bacteria expressing *pept-1* dsRNA, and imaged by bright-field microscopy on day 1 of adulthood. WT: wild-type N2 (A – Hansen lab, B – Tuck lab), *rrf-1(pk1417).4x: rrf-1(pk1417)* outcrossed 4 times to WT(A). (**A**) The *rrf-1* animals were smaller and have a clear appearance, whereas the wild-type and hypersensitive RNAi strain *rrf-3* were developmentally delayed. (**B**) Quantification of the mid-body diameter of all the strains shows that all strains are significantly smaller than the unaffected *rde-1* strain. This experiment has been repeated three times with similar results. Size quantification was determined with 7–10 worms per strain. Bars show the mean + SEM of one representative experiment. P<0.0001 was determined with one-way ANOVA, and individual significance was determined with Bonferroni’s multiple comparison test: **P<0.005 ***P<0.0005.
(TIF)

Figure S4 ***rrf-1*** **mutants are partially affected by RNAi targeting the hypodermis.** (**A**) The indicated *C. elegans* strains were raised on bacteria expressing *bli-3* (expressed in the hypodermis), *bli-4* (expressed in the hypodermis, intestine, vulva, and ventral nerve cords) or *die-1* (expressed embryonically in the hypodermis, pharyngeal cells, and muscle/gut primordium precursor cells) RNAi from hatching. Worms were assayed for the indicated phenotypes on day 1 of adulthood of the first generation for *bli-3* and *bli-4* RNAi and the second generation of RNAi treatment for *die-1* RNAi. These assays have been repeated two or three times with similar results. WT: wild-type N2 (A – Hansen lab, B – Tuck lab), *rrf-1(pk1417).4x: rrf-1(pk1417)* outcrossed 4 times to WT(A). Phenotype abbreviations: Bli: blistered cuticle; Bmd: body morphology defects. n.a.: not assayed in this experiment, but these strains showed no effect when ∼10 worms were imaged in a separate experiment (data not shown). †: phenotypes only observed in 2nd generation of RNAi treatment. (**B**) The indicated *C. elegans* strains were raised on bacteria expressing *bli-4* dsRNA, and imaged by bright-field microscopy on day 1 of adulthood. The gene *bli-4* is expressed in the hypodermis, intestine, vulva, and ventral nerve cords. All strains except the RNAi-resistant *rde-1(ne219)* strain were affected by the RNAi treatment. This experiment has been repeated twice with similar results. (**C**) Quantification of the fluorescence intensity of wild-type and *rrf-1(pk1417)* animals expressing *SCMp*::*gfp* raised on control RNAi and *gfp* RNAi. Data are from images of one representative experiment (**Figure 3B**). Because the transgene expression in the *rrf-1* strain background was greatly increased compared to the wild-type animals, the exposure times were 5 ms and 100 ms, respectively. Student’s *t*-test was performed for statistical analysis: *P<0.05, ***P<0.0005.
(TIF)

Figure S5 ***rrf-1*** **mutants are inefficient in processing** ***gfp*** **RNAi in the somatic gonad.** The indicated *C. elegans* strains were raised on bacteria expressing *gfp* dsRNA, and imaged by DIC and fluorescence microscopy (overlays shown here) on day 1 of adulthood. *rrf-1* mutants carried the *pk1417* allele. (**A**) The *lag-2p::gfp* reporter is expressed solely in the distal tip cell (DTC) of *C. elegans*. GFP expression in this strain was dramatically reduced upon treatment with *gfp* RNAi, whereas the *rrf-1* mutant maintained DTC fluorescence. DTCs are indicated by arrows. Asterisk denotes the DTC from the opposite gonad arm, which is out of focus in this image. The exposure time for the GFP channel was 100 ms. (**B**) The *lgg-1p::gfp::lgg-1* reporter is expressed in the intestine, pharynx, somatic gonad, and hypodermis. GFP expression in this strain was dramatically reduced upon treatment with *gfp* RNAi. The *rrf-1* mutant lost all intestinal GFP fluorescence but maintained GFP in the spermatheca (SP) and vulva. The spermatheca is indicated by an arrow. The exposure time for the GFP channel was 5 ms. (**C**) Quantification of the fluorescence intensity of animals expressing *lag-2p::gfp* raised on OP50 bacteria from images of one representative experiment. Student’s *t*-test was performed for statistical analysis: **P<0.005 (similar results were obtained when empty-vector control bacteria were used as the control condition, data not shown). The GFP intensity of the *rrf-1* strain is under-represented due to saturation of the GFP level. This experiment was repeated twice with similar results. (**D**) Quantification of the fluorescence intensity of images of whole animals expressing *lgg-1p::gfp::lgg-1* raised on OP50 bacteria from images of one representative experiment. Student’s *t*-test was performed for statistical analysis: n.s., P>0.05 (similar results were obtained when empty-vector control bacteria were used as the control condition, data not shown). This experiment was repeated three times with similar results (See also **Figure 2A**).
(TIF)

Figure S6 ***rrf-1*** **mutants do not show deletions or genomic rearrangements in the** ***eri-6/eri-7*** **gene locus.** Image of an ethidium-bromide stained agarose gel after electrophoresis of PCR-amplified DNA of the indicated *C. elegans* strains, with primer combination P1 + P2 or P1 + P3. In wild-type animals the genes *eri-6* and *eri-7* are in the *trans* position, and *eri-6* is flanked by direct repeats (indicated by blue arrows). Mutation *mg441* induces the inversion of the direct repeats, which leads to a genomic rearrangement of *eri-6* into the *cis* position. The assay was designed to detect a possible genomic rearrangement as indicated by the presence of an amplification product with primer pair P1 + P3 instead of P1 + P2. PCR with P1 + P2 of all strains resulted in the same size amplification product, indicating that none of the strains carries a deletion mutation at this gene locus. WT: wild-type N2 (A – Hansen lab, B – Tuck lab), *rrf-1(pk1417).4x: rrf-1(pk1417)* outcrossed 4 times to WT(A).
(TIF)

Figure S7 ***rrf-1*** **mutants display no increase in** ***daf-16*** **mRNA levels, but increased transgenic expression of the** ***daf-16*** **target gene** ***sod-3.*** (**A**) The indicated *C. elegans* strains were raised on OP50 bacteria and imaged by bright-field and fluorescence microscopy (overlays shown here) on day 1 of adulthood. *rrf-1(pk1417)* mutants have increased GFP expression as shown in representative images. The exposure time for the GFP channel was 200 ms. (**B**) Quantification of the intestinal fluorescence intensity of animals expressing *daf-16p::gfp::daf-16* from images of one representative experiment. Student’s *t*-test was performed for statistical analysis: ***P<0.0005. This experiment has been repeated three times with similar results (see also **Figure 2B**). (**C**) The *daf-16 *mRNA levels of a mixed population of wild-type (WT, N2(A)), *rrf-1(pk1417).4x* (*rrf-1(pk1417)* mutant outcrossed four times to N2(A)), and the reporter strains expressing *daf-16p::gfp::daf-16* and *rrf-1; daf-16p::gfp::daf-16* were determined. Bars show the mean + SEM of three independent experiments; n.s.: P>0.05 (one-way ANOVA). (**D**) The indicated *C. elegans* strains were raised on OP50 bacteria and imaged on day 3 of adulthood. *rrf-1* mutants have increased GFP expression as shown in representative images. The exposure time for the GFP channel was 200 ms. (**E**) Quantification of the intestinal fluorescence intensity of animals expressing *sod-3p::gfp* from images of one representative experiment. Student’s *t*-test was performed for statistical analysis: ***P<0.0005. This experiment has been repeated three times with similar results. (**F**) Three independent lifespan experiments comparing wild-type (WT(A): N2 from the Hansen lab) to *rrf-1(pk1417).4x* animals (*rrf-1(pk1417)* mutant outcrossed 4 times to N2(A)) were performed at 20°C using OP50 bacteria as the food source. MLS: mean lifespan; N: population number; P-value obtained with Mantel-Cox log-rank test.
(TIF)
