# Supplementary figures and images for: C. elegans rrf-1 Mutations Maintain RNAi Efficiency in the Soma in Addition to the Germline
Source: PLoS One. 2012 May 4;7(5):e35428. doi: 10.1371/journal.pone.0035428 (PMC3344830; doi:10.1371/journal.pone.0035428)

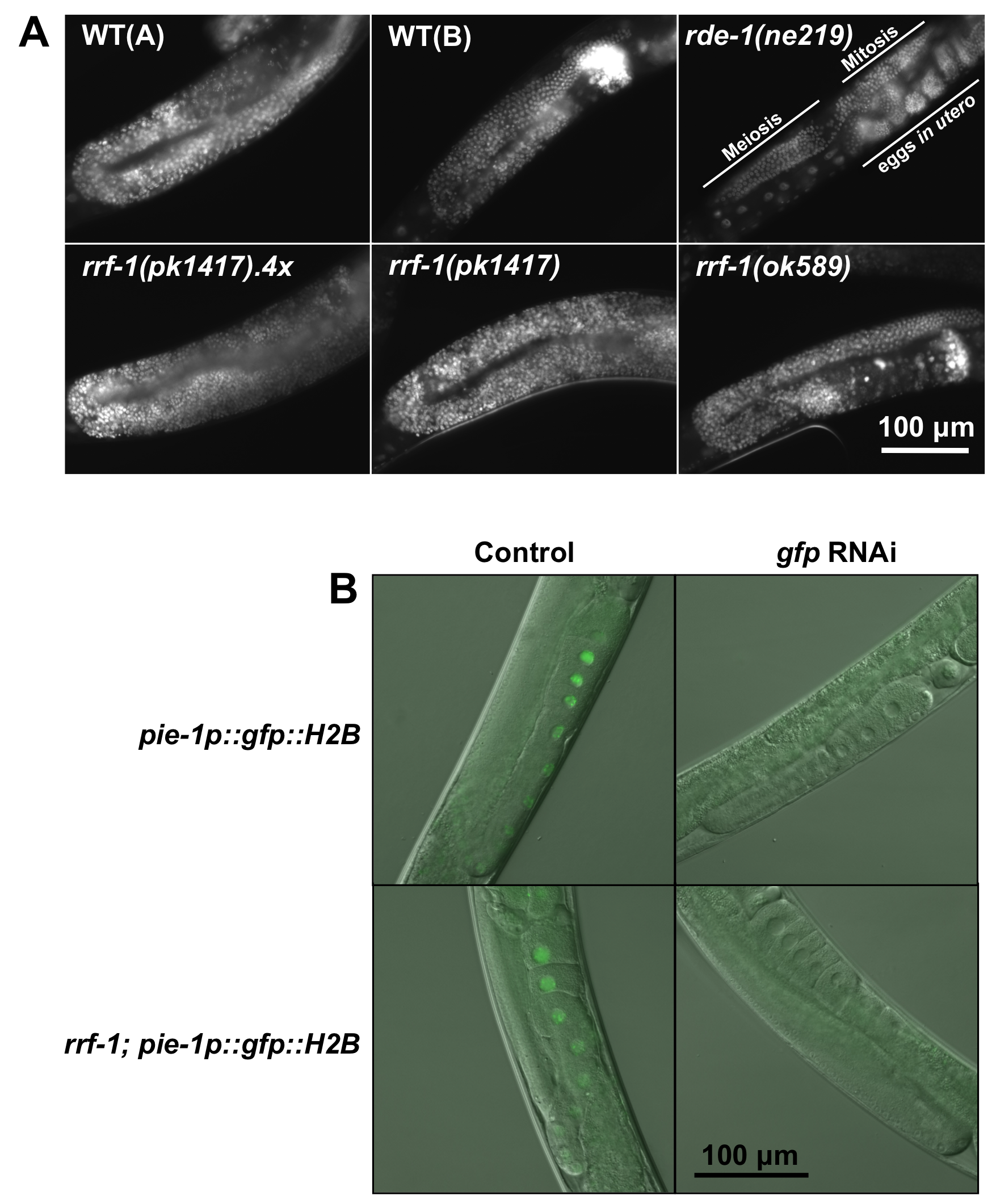

Supplement: Figure S1 — rrf-1 mutants are capable of processing RNAi in the germline. (A) The indicated C. elegans strains were raised on bacteria expressing gld-1 dsRNA, fixed in methanol on day 3 of adulthood, and subsequently stained with DAPI and imaged by fluorescence microscopy. All strains display mitotic germ cell overproliferation except the RNAi-resistant rde-1 strain, which shows typical mitotic and meiotic zones in the distal end of the gonad, and eggs in utero in the proximal part of the gonad. This experiment was repeated twice with 50–100 worms per strain with similar results. WT: wild-type N2 (A – Hansen lab, B – Tuck lab), rrf-1(pk1417).4x: rrf-1(pk1417) outcrossed 4 times to WT(A). (B) The indicated C. elegans strains were raised on bacteria expressing gfp dsRNA and imaged by DIC and fluorescence microscopy (overlays shown here) on day 1 of adulthood. rrf-1 mutants carried the pk1417 allele. The pie-1p::gfp::H2B reporter is expressed in the germline of C. elegans. GFP expression is completely abolished upon treatment with gfp RNAi, in both the wild-type and rrf-1 backgrounds. The image shows one arm of the gonad including oocytes, and was acquired with an exposure time of 100 ms. This experiment was repeated twice with 30–50 worms per strain and imaging of ∼10 per experiment, with similar results. (TIF) [file pone.0035428.s001.tif]

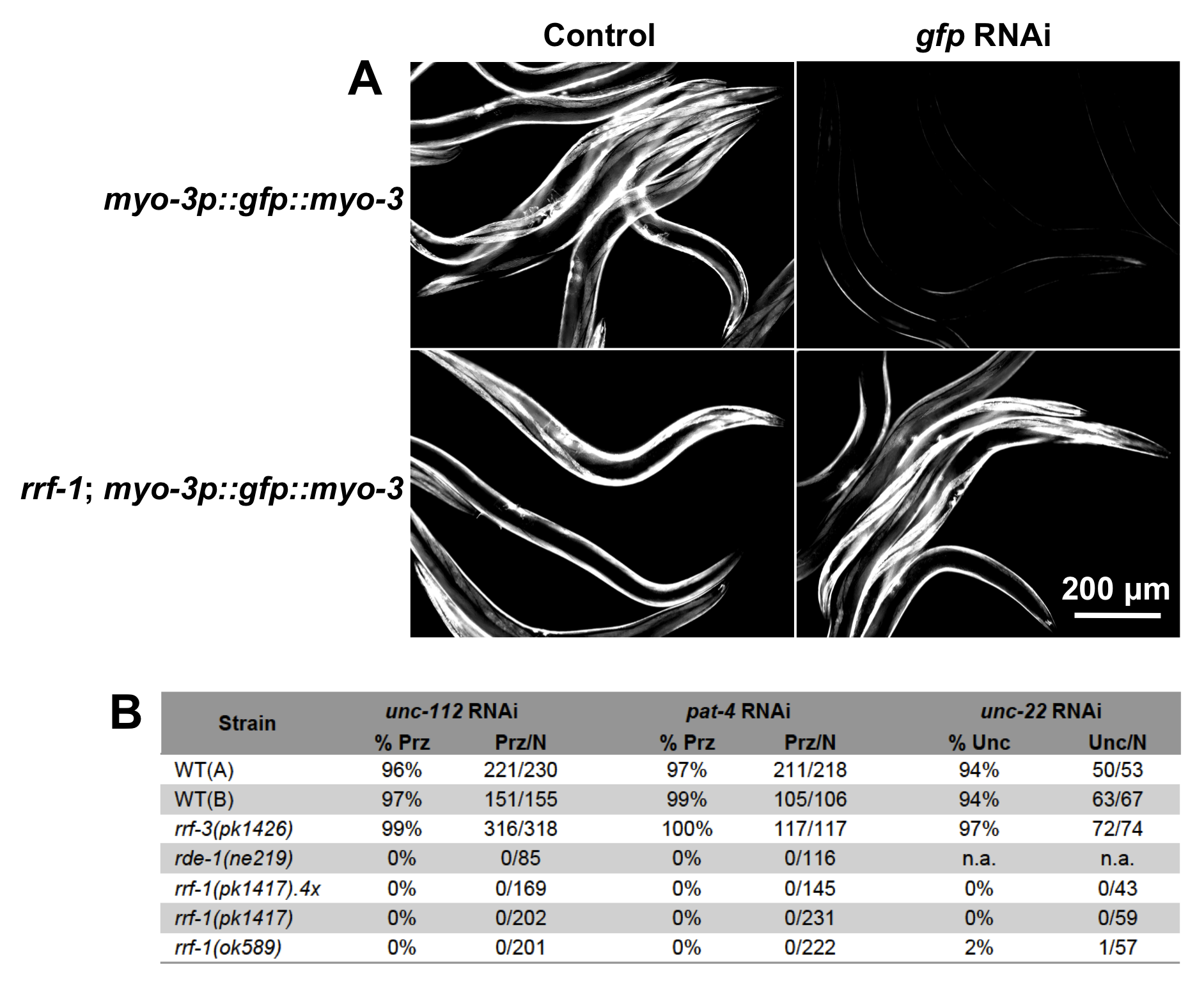

Supplement: Figure S2 — rrf-1 mutants are resistant to RNAi in the muscle. (A) The indicated C. elegans strains were raised on bacteria expressing gfp dsRNA, and imaged by fluorescence microscopy on day 1 of adulthood. rrf-1 mutants carried the pk1417 allele. The body-wall muscle-specific GFP expression is maintained in the rrf-1 background, but completely abolished in the wild-type background. The exposure time for the GFP channel was 200 ms. This experiment was repeated twice with similar results. (B) The indicated C. elegans strains were raised from hatching on bacteria expressing muscle-specific unc-112, pat-4, and unc-22 RNAi and were assayed for paralysis and twitching on day 1 of adulthood. The experiments have been repeated at least two times with similar results. WT: wild-type N2 (A – Hansen lab, B – Tuck lab), rrf-1(pk1417).4x: rrf-1(pk1417) outcrossed 4 times to WT(A). Phenotype abbreviations: Prz: paralyzed; Unc: uncoordinated, sub-class, Twitcher. n.a.: not assayed. (TIF) [file pone.0035428.s002.tif]

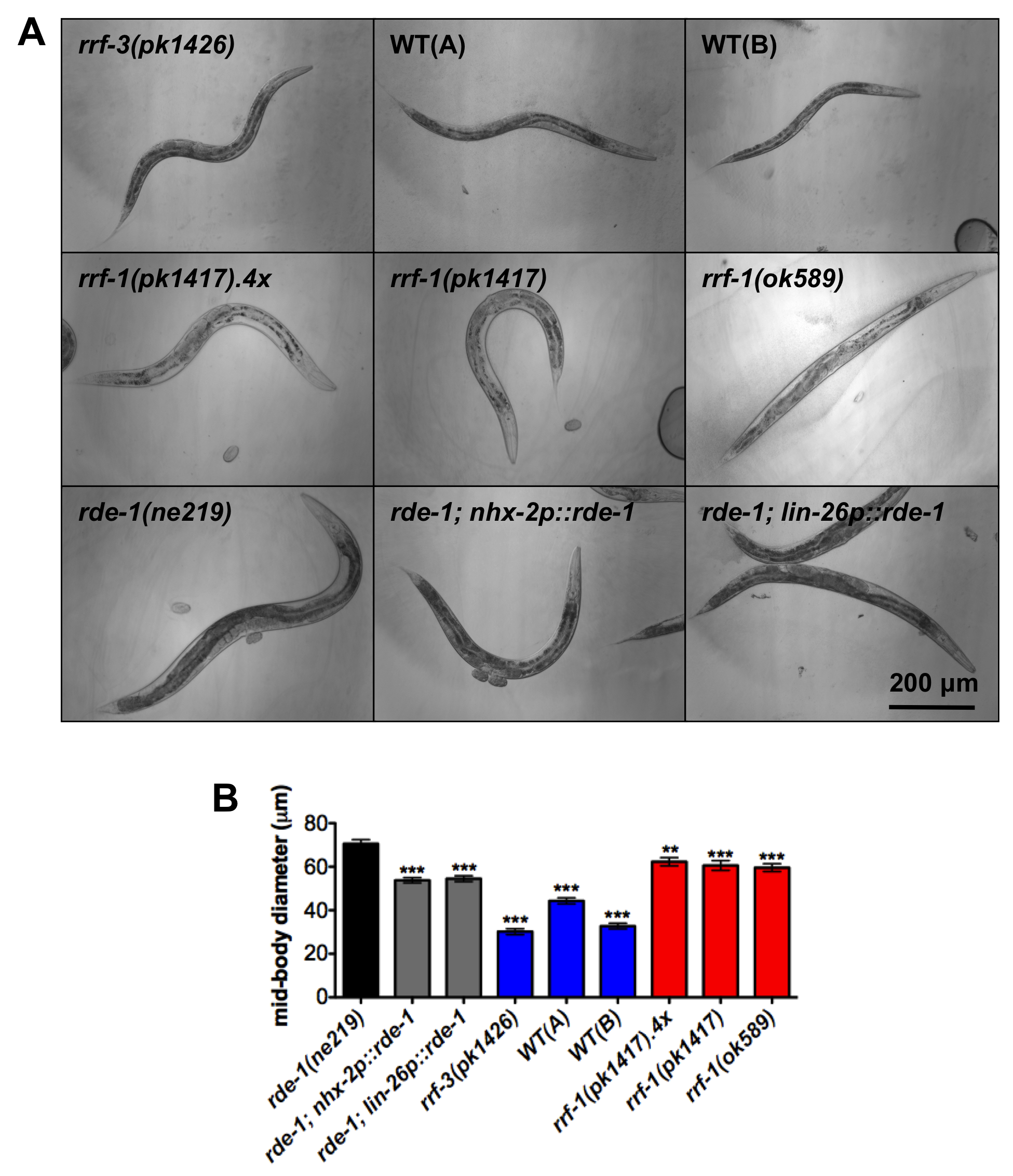

Supplement: Figure S3 — Intestinal pept-1 RNAi induces phenotypes in rrf-1 mutants. The indicated C. elegans strains were raised on bacteria expressing pept-1 dsRNA, and imaged by bright-field microscopy on day 1 of adulthood. WT: wild-type N2 (A – Hansen lab, B – Tuck lab), rrf-1(pk1417).4x: rrf-1(pk1417) outcrossed 4 times to WT(A). (A) The rrf-1 animals were smaller and have a clear appearance, whereas the wild-type and hypersensitive RNAi strain rrf-3 were developmentally delayed. (B) Quantification of the mid-body diameter of all the strains shows that all strains are significantly smaller than the unaffected rde-1 strain. This experiment has been repeated three times with similar results. Size quantification was determined with 7–10 worms per strain. Bars show the mean + SEM of one representative experiment. P<0.0001 was determined with one-way ANOVA, and individual significance was determined with Bonferroni’s multiple comparison test: **P<0.005 ***P<0.0005. (TIF) [file pone.0035428.s003.tif]

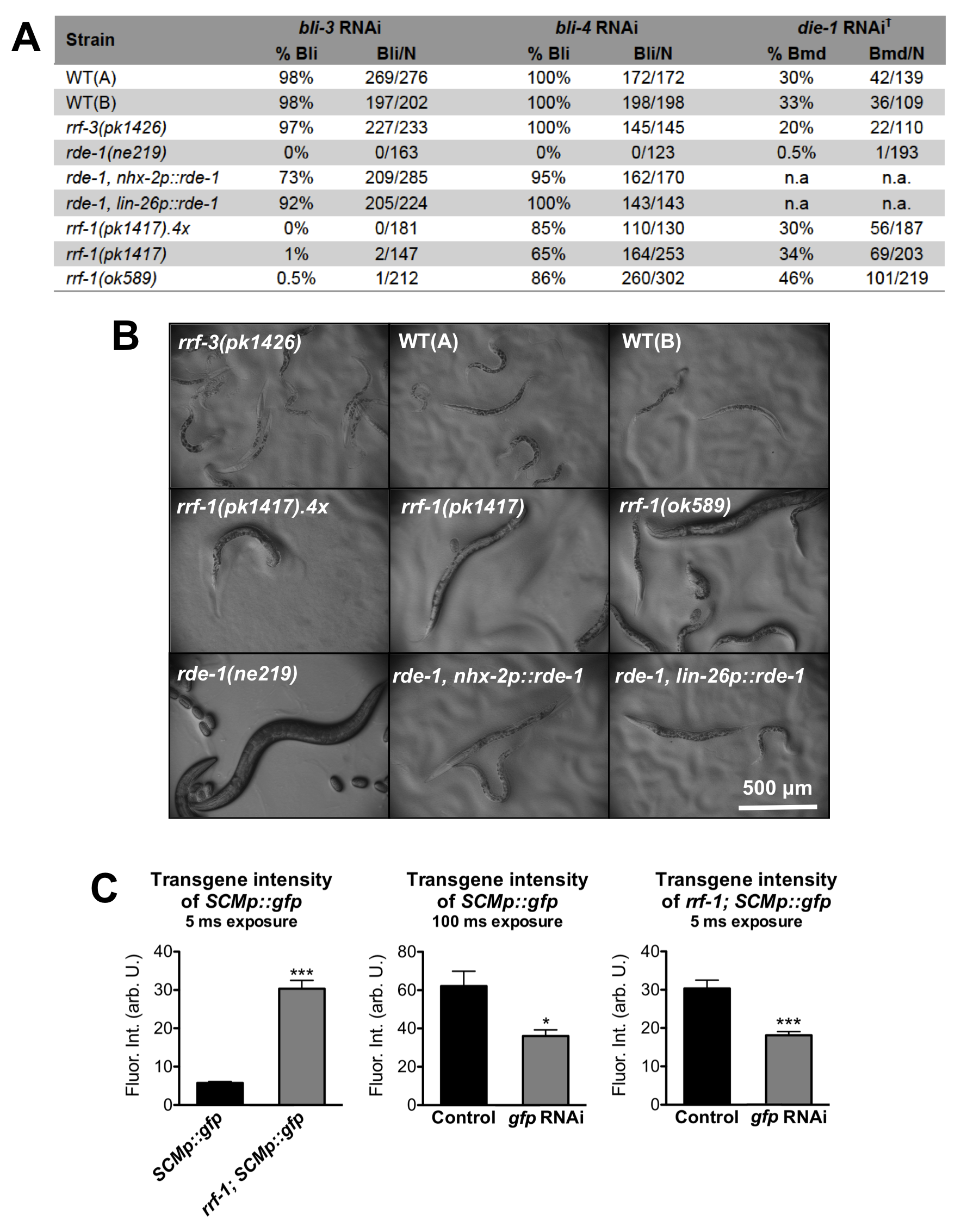

Supplement: Figure S4 — rrf-1 mutants are partially affected by RNAi targeting the hypodermis. (A) The indicated C. elegans strains were raised on bacteria expressing bli-3 (expressed in the hypodermis), bli-4 (expressed in the hypodermis, intestine, vulva, and ventral nerve cords) or die-1 (expressed embryonically in the hypodermis, pharyngeal cells, and muscle/gut primordium precursor cells) RNAi from hatching. Worms were assayed for the indicated phenotypes on day 1 of adulthood of the first generation for bli-3 and bli-4 RNAi and the second generation of RNAi treatment for die-1 RNAi. These assays have been repeated two or three times with similar results. WT: wild-type N2 (A – Hansen lab, B – Tuck lab), rrf-1(pk1417).4x: rrf-1(pk1417) outcrossed 4 times to WT(A). Phenotype abbreviations: Bli: blistered cuticle; Bmd: body morphology defects. n.a.: not assayed in this experiment, but these strains showed no effect when ∼10 worms were imaged in a separate experiment (data not shown). †: phenotypes only observed in 2nd generation of RNAi treatment. (B) The indicated C. elegans strains were raised on bacteria expressing bli-4 dsRNA, and imaged by bright-field microscopy on day 1 of adulthood. The gene bli-4 is expressed in the hypodermis, intestine, vulva, and ventral nerve cords. All strains except the RNAi-resistant rde-1(ne219) strain were affected by the RNAi treatment. This experiment has been repeated twice with similar results. (C) Quantification of the fluorescence intensity of wild-type and rrf-1(pk1417) animals expressing SCMp::gfp raised on control RNAi and gfp RNAi. Data are from images of one representative experiment ( Figure 3B ). Because the transgene expression in the rrf-1 strain background was greatly increased compared to the wild-type animals, the exposure times were 5 ms and 100 ms, respectively. Student’s t-test was performed for statistical analysis: *P<0.05, ***P<0.0005. (TIF) [file pone.0035428.s004.tif]

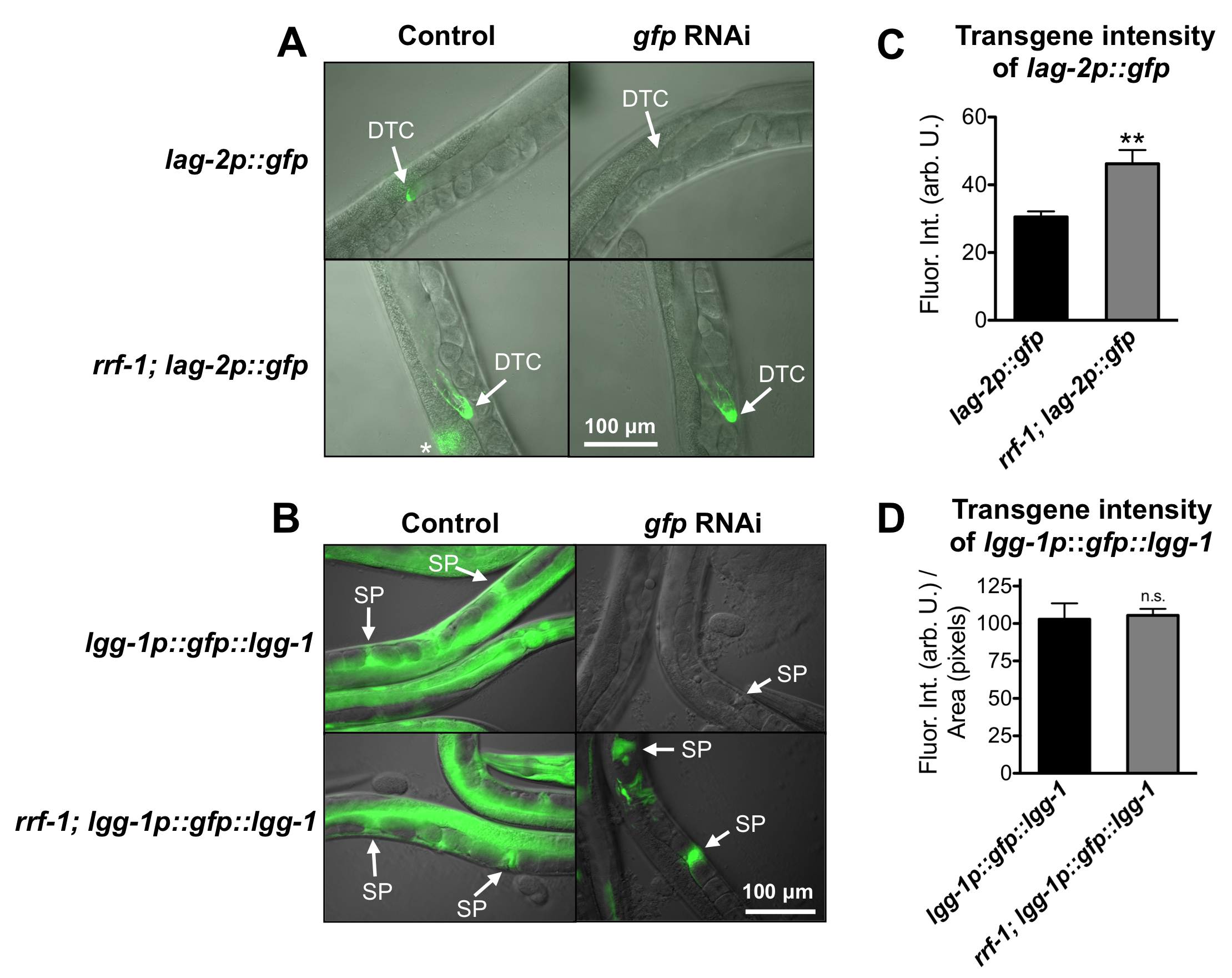

Supplement: Figure S5 — rrf-1 mutants are inefficient in processing gfp RNAi in the somatic gonad. The indicated C. elegans strains were raised on bacteria expressing gfp dsRNA, and imaged by DIC and fluorescence microscopy (overlays shown here) on day 1 of adulthood. rrf-1 mutants carried the pk1417 allele. (A) The lag-2p::gfp reporter is expressed solely in the distal tip cell (DTC) of C. elegans. GFP expression in this strain was dramatically reduced upon treatment with gfp RNAi, whereas the rrf-1 mutant maintained DTC fluorescence. DTCs are indicated by arrows. Asterisk denotes the DTC from the opposite gonad arm, which is out of focus in this image. The exposure time for the GFP channel was 100 ms. (B) The lgg-1p::gfp::lgg-1 reporter is expressed in the intestine, pharynx, somatic gonad, and hypodermis. GFP expression in this strain was dramatically reduced upon treatment with gfp RNAi. The rrf-1 mutant lost all intestinal GFP fluorescence but maintained GFP in the spermatheca (SP) and vulva. The spermatheca is indicated by an arrow. The exposure time for the GFP channel was 5 ms. (C) Quantification of the fluorescence intensity of animals expressing lag-2p::gfp raised on OP50 bacteria from images of one representative experiment. Student’s t-test was performed for statistical analysis: **P<0.005 (similar results were obtained when empty-vector control bacteria were used as the control condition, data not shown). The GFP intensity of the rrf-1 strain is under-represented due to saturation of the GFP level. This experiment was repeated twice with similar results. (D) Quantification of the fluorescence intensity of images of whole animals expressing lgg-1p::gfp::lgg-1 raised on OP50 bacteria from images of one representative experiment. Student’s t-test was performed for statistical analysis: n.s., P>0.05 (similar results were obtained when empty-vector control bacteria were used as the control condition, data not shown). This experiment was repeated three times with similar results (Se [file pone.0035428.s005.tif]

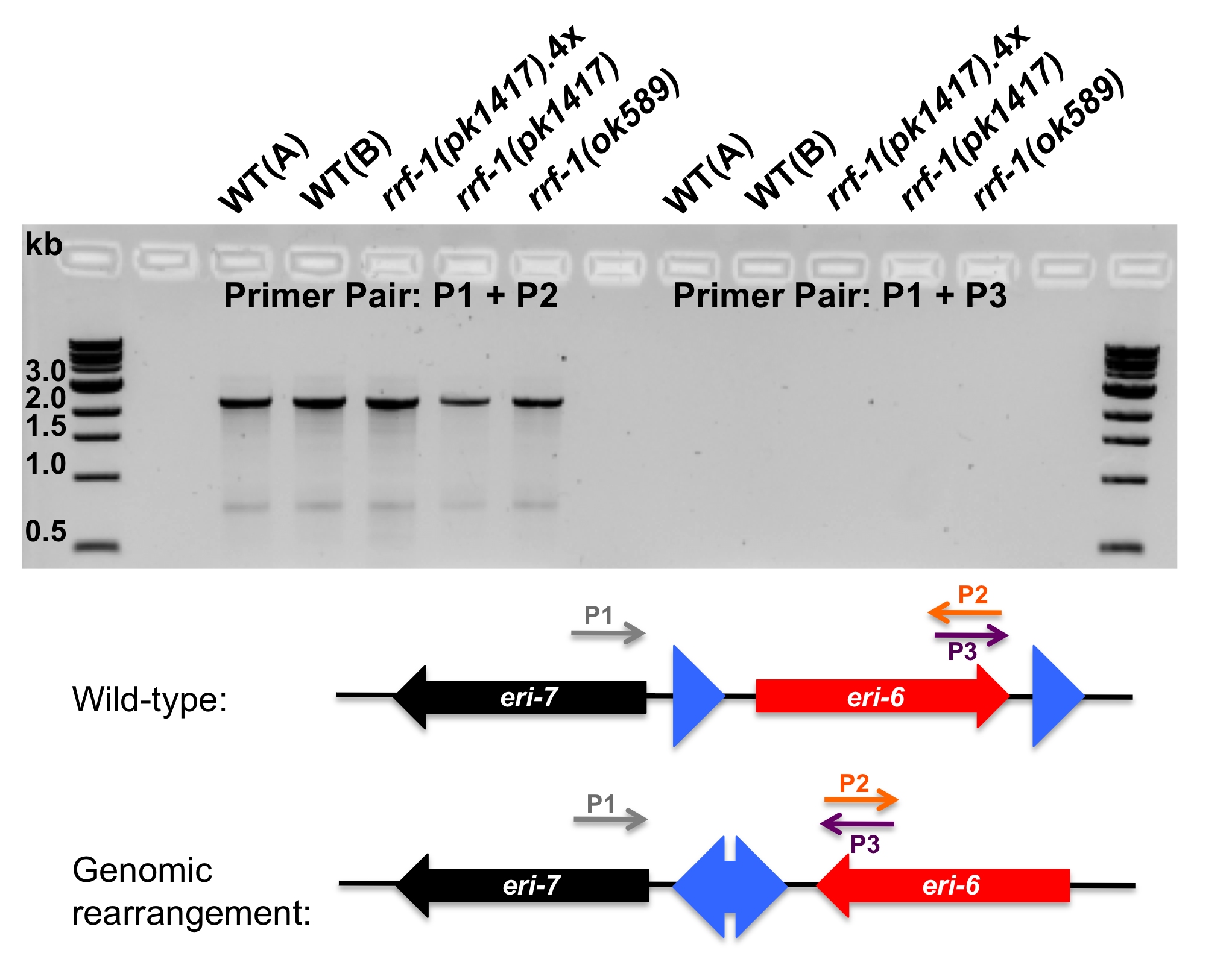

Supplement: Figure S6 — rrf-1 mutants do not show deletions or genomic rearrangements in the eri-6/eri-7 gene locus. Image of an ethidium-bromide stained agarose gel after electrophoresis of PCR-amplified DNA of the indicated C. elegans strains, with primer combination P1 + P2 or P1 + P3. In wild-type animals the genes eri-6 and eri-7 are in the trans position, and eri-6 is flanked by direct repeats (indicated by blue arrows). Mutation mg441 induces the inversion of the direct repeats, which leads to a genomic rearrangement of eri-6 into the cis position. The assay was designed to detect a possible genomic rearrangement as indicated by the presence of an amplification product with primer pair P1 + P3 instead of P1 + P2. PCR with P1 + P2 of all strains resulted in the same size amplification product, indicating that none of the strains carries a deletion mutation at this gene locus. WT: wild-type N2 (A – Hansen lab, B – Tuck lab), rrf-1(pk1417).4x: rrf-1(pk1417) outcrossed 4 times to WT(A). (TIF) [file pone.0035428.s006.tif]

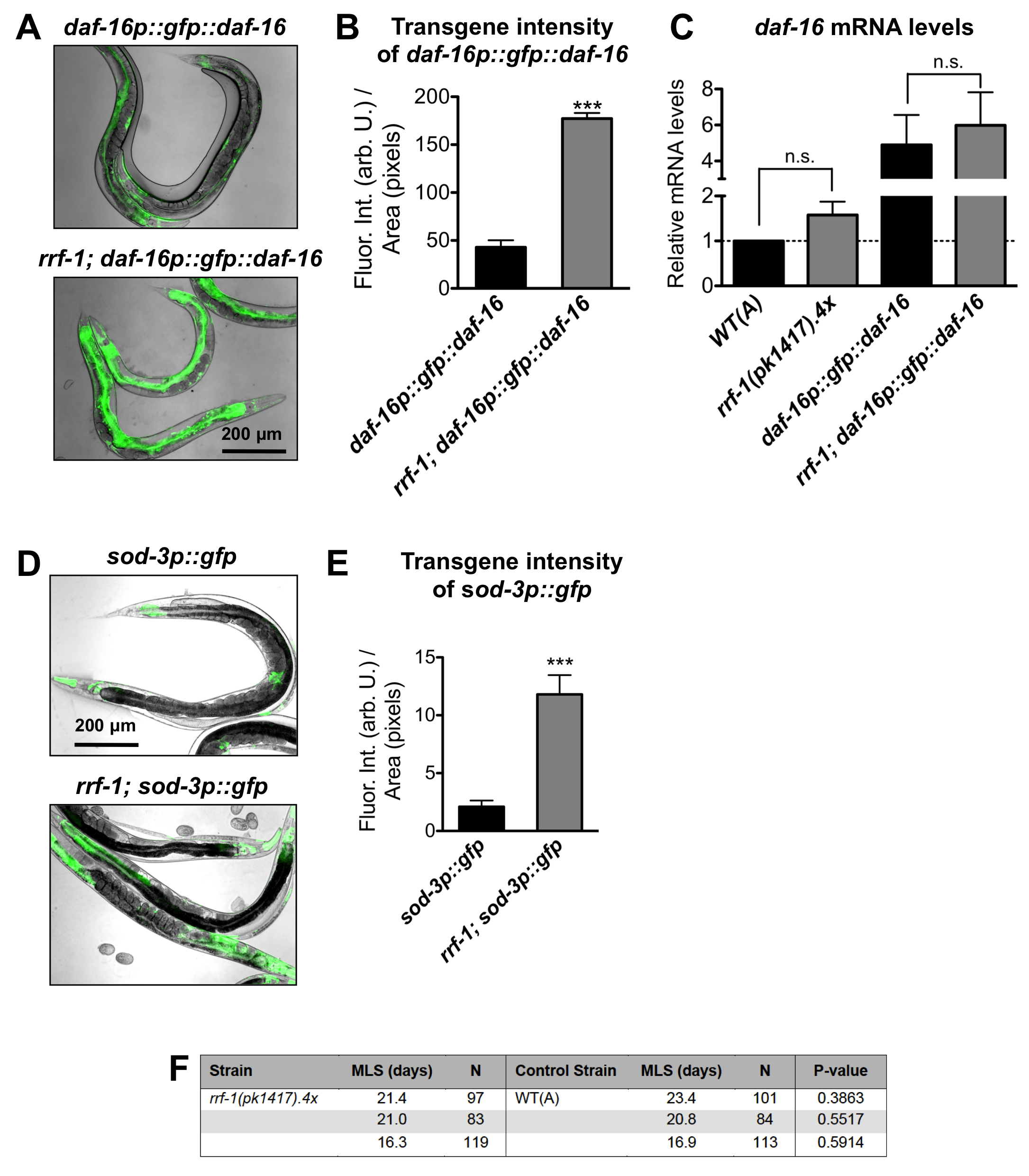

Supplement: Figure S7 — rrf-1 mutants display no increase in daf-16 mRNA levels, but increased transgenic expression of the daf-16 target gene sod-3. (A) The indicated C. elegans strains were raised on OP50 bacteria and imaged by bright-field and fluorescence microscopy (overlays shown here) on day 1 of adulthood. rrf-1(pk1417) mutants have increased GFP expression as shown in representative images. The exposure time for the GFP channel was 200 ms. (B) Quantification of the intestinal fluorescence intensity of animals expressing daf-16p::gfp::daf-16 from images of one representative experiment. Student’s t-test was performed for statistical analysis: ***P<0.0005. This experiment has been repeated three times with similar results (see also Figure 2B ). (C) The daf-16 mRNA levels of a mixed population of wild-type (WT, N2(A)), rrf-1(pk1417).4x (rrf-1(pk1417) mutant outcrossed four times to N2(A)), and the reporter strains expressing daf-16p::gfp::daf-16 and rrf-1; daf-16p::gfp::daf-16 were determined. Bars show the mean + SEM of three independent experiments; n.s.: P>0.05 (one-way ANOVA). (D) The indicated C. elegans strains were raised on OP50 bacteria and imaged on day 3 of adulthood. rrf-1 mutants have increased GFP expression as shown in representative images. The exposure time for the GFP channel was 200 ms. (E) Quantification of the intestinal fluorescence intensity of animals expressing sod-3p::gfp from images of one representative experiment. Student’s t-test was performed for statistical analysis: ***P<0.0005. This experiment has been repeated three times with similar results. (F) Three independent lifespan experiments comparing wild-type (WT(A): N2 from the Hansen lab) to rrf-1(pk1417).4x animals (rrf-1(pk1417) mutant outcrossed 4 times to N2(A)) were performed at 20°C using OP50 bacteria as the food source. MLS: mean lifespan; N: population number; P-value obtained with Mantel-Cox log-rank test. (TIF) [file pone.0035428.s007.tif]
